# A descriptive study on oral hygiene practice and caries increment in children with growth stunting

**DOI:** 10.3389/froh.2023.1236228

**Published:** 2023-11-06

**Authors:** Tri Nugrahaeni Putri, Ratna Indriyanti, Arlette Suzy Setiawan

**Affiliations:** ^1^ Dental Education Program, Faculty of Dentistry, Universitas Padjadjaran, Bandung, Indonesia; ^2^ Department of Pediatric Dentistry, Faculty of Dentistry, Universitas Padjadjaran, Bandung, Indonesia

**Keywords:** stunting, caries increment, oral hygiene practice, malnutrition (MeSH), child-age

## Abstract

**Introduction:**

Stunting is a condition of malnutrition in children from the womb to the early life stage that causes growth failure in the body and brain. Stunting influences the development and integrity of the oral cavity and increases the risk of developing diseases in the oral cavity, such as dental caries. The growth barriers in stunting children and parental knowledge can affect maintaining oral hygiene. This study aims to determine the description of oral hygiene practice and caries increment in children with growth stunting.

**Methods:**

This type of research is a quantitative descriptive with a Secondary Data Analysis approach in the form of examination results for the presence or absence of caries through the ICDAS index and the results of the oral hygiene practice questionnaire on 113 children with growth stunting in Sukajadi district, Bandung.

**Results:**

Most stunting children (60.2%) experienced increased caries rates classified as low, and 70.8% had poor oral hygiene practices. There were 50 children (44.3%) with poor oral hygiene practice with low caries increment, while 18 children (15.9%) had good oral hygiene practice with low caries increment.

**Conclusion:**

Oral hygiene practice is classified as poor, but caries increment is still relatively low in most stunting children in Sukajadi district, Bandung.

## Introduction

The Ministry of Health of the Republic of Indonesia declared that the prevalence of stunting in Indonesia reached 29.6% in 2017 (1), exceeding the threshold set by the World Health Organization (WHO) of 20% (2). Supported by data from Basic Health Research, the prevalence of stunting among children under five in Indonesia remains significantly high (3). In 2013, the rates were 37.2% (18% severely stunted and 19.2% moderately stunted). In 2018, the rates were 30.8% (11.5% severely stunted and 19.3% moderately stunted), indicating that approximately one-third of children in Indonesia have poor nutritional status (1). In addition, the Indonesian Child Nutrition Status Survey (SSGBI) in 2019 reported a stunting prevalence of 26.2% in West Java Province, while the Indonesian Nutrition Status Survey (SSGI) in 2021 recorded a rate of 24.5% for the same region (4).

Stunting is a chronic nutritional deficiency characterised by short stature, measured by height-for-age indicators (HAZ). The parameter assesses linear growth in children under two years of age to determine stunting status (1). According to the WHO, stunting is when a child's height falls below two standard deviations (SD) from the median standard growth (2, 5). Scientifically, stunting is a nutritional deficiency that occurs from early life stages, including the prenatal period, leading to growth failure in both the body and brain (6). Other characteristics of stunted children include delayed height and weight growth, age-appropriate dental growth, and motor development (7). Poor motor skills in children with long-term malnutrition are associated with delayed maturation processes and progressive muscle function impairment (8). Stunting is irreversible due to insufficient nutrition and repeated infections during the first 1,000 days of life. Given this phenomenon, stunting has become a significant public health concern with negative impacts on various aspects, including increased disease risks, hindered motor and mental development, and even mortality if not addressed (5).

In dentistry, stunting influences the development and integrity of the oral cavity, increasing the risk of oral diseases (9, 10). The most common oral disease is dental caries, indicating a decline in oral health and its correlation with stunted children. Nutritional disturbances in stunting can cause hyposalivation, contributing to dental caries. Reduced saliva flow decreases saliva buffering and self-cleansing capacity, making the oral environment more susceptible to bacterial colonisation (9). Additionally, enamel developmental defects predispose factors for high caries in children with nutritional disorders, as the teeth become more vulnerable to cariogenic bacteria (9, 11, 12). A longitudinal study conducted by Delgado-Angulo in Peru showed that stunted children exhibited a significant increase in caries compared to normal children during a three-and-a-half-year follow-up (13).

Dental caries involves the interaction between acid-producing microorganisms, tooth surfaces, and substrates over time. Clinical assessment systems such as the International Caries Detection and Assessment System (ICDAS) or the DMF-T and def-t indexes issued by the WHO can detect and evaluate caries activity (14, 15). Consistent with Rahman's study in 2016, the def-t index was significantly higher in stunted children compared to non-stunted children, highlighting the urgency of addressing dental caries in stunted children and the importance of preventive programs to reduce the risk of caries progression in this population (16).

The data highlights the urgency of addressing dental caries in stunted children and emphasises the importance of preventive programs to reduce the risk of caries progression in this population. One way to address the significant increase in caries is by implementing optimal oral hygiene practices. Oral hygiene practices encompass various efforts or behaviours to maintain oral health and cleanliness, aiming to prevent dental and oral diseases that can impact an individual's productivity and quality of life (17, 18). The motor development barriers in stunted children can influence their oral hygiene practices (7). Parental knowledge also plays a crucial role in shaping the oral hygiene behaviours of children (6). Good oral hygiene practices include appropriate frequency of tooth brushing per day, determining the optimal time for tooth brushing, consumption of tooth-friendly foods, and regular dental check-ups, which should be initiated and habituated from an early age (17, 18). This study aims to determine the description of oral hygiene practices and caries increment in stunted children.

## Material and methods

A quantitative descriptive study with a Secondary Data Analysis (SDA) approach with the secondary data retrieved from the documentation of the Academic Leadership Grant (ALG) study titled “Dental Health Aspects and Family Approaches in Stunting Prevention” in Sukajadi Sub-District, Bandung City, from 2020 to 2022. The population in this study consists of 113 participants (aged 1–3 years old and the different stages of tooth eruption and primary molar presence), based on secondary data, including the examination results for the presence or absence of caries using the ICDAS index and the Oral Hygiene Practice questionnaire results in children in Sukajadi Sub-District, Bandung City. As for the oral hygiene index, we use the debris index (19). This component assesses the presence of soft debris, such as food particles and plaque, on tooth surfaces. It is scored based on a scale that ranges from 0 to 3:

• 0: No debris or stain present.
• 1: Soft debris covering not more than one-third of the tooth surface.
• 2: Soft debris covering more than one-third but not more than two-thirds of the tooth surface.
• 3: Soft debris covering more than two-thirds of the tooth surface.

In the realm of oral hygiene practices, one's performance can be categorized into two distinct categories. When the total score obtained by respondents surpasses the overall mean score, their oral hygiene practice is deemed commendable and classified as “good.” Conversely, if the total score acquired by respondents falls short of the overall mean score, their oral hygiene practice is labeled as “poor.” This clear-cut distinction helps assess and gauge the effectiveness of oral hygiene routines, promoting awareness and better practices for maintaining dental health (20).

The sampling technique used in this study is total sampling, where all population members are included as samples. The inclusion criteria for this study are children with a birth length of ≤50 cm and participants with complete data, while the exclusion criteria are participants with incomplete data or questionnaires. The study was conducted from January to February 2023.

The research instrument includes Microsoft Excel software, an SPSS application, and secondary data such as oral hygiene practice questionnaires and caries examination results in stunted children from 2020 to 2022. The accumulated scores of appropriate answers in the oral hygiene practice questionnaire were grouped into two categories: good if the respondent's score is greater than or equal to the mean and poor if the respondent's score is less than the mean. The caries examination results, representing caries increment from the first to the third year, were grouped into two categories: low if the respondent's score is less than the mean and high if the respondent's score is greater than or equal to the mean. The complete and organised data were coded in SPSS and then analysed using descriptive statistical tests. This technique was used to examine frequencies, percentages, and cross-tabulations. The results of the data analysis were presented in tabular form. This study has obtained ethical approval from the Research Ethics Committee of Universitas Padjadjaran, Bandung, with document number 134/UN6.KEP/EC/2022.

## Results

The research findings were obtained from a sample of 113 children, and the oral hygiene practices, consisting of eight components, can be seen in Table 1, which shows that out of 113 participants, data were obtained as follows: 71 children (62.8%) brush their teeth regularly every day, all participants (100%) have their toothbrush, 67 children (59.3%) use fluoride toothpaste when brushing their teeth, and 70 children (61.9%) still consume breast milk or milk with a bottle at night. Furthermore, 78 children (69%) brush their teeth for less than 2 min, 100 children (88.5%) only brush their teeth once a day, 56 children (49.6%) only brush their teeth in the afternoon, and 64 children (56.6%) brush their teeth using horizontal movements. The data on the rate of caries increment in stunted children from the first to the third year can be seen in Table 2.

**Table 1:** Distribution of oral hygiene practice questionnaire results among stunted children in Sukajadi sub-district, Bandung city.

No			F	%
1	Regularly tooth brushing	Yes	71	62.8
		No	42	37.2
2	Own a private tooth brush	Yes	113	100
		No	0	0
3	Using tooth paste	Yes	67	59.3
		No	46	40.7
4	Sleep at night with a pacifier or breast milk	Yes	70	61.9
		No	43	38.1
5	Tooth brushing duration	2 min	35	31
		<2 min	78	69
6	Frequency of brushing teeth in a day	1	100	88.5
		2	12	10.6
		Uncertain	1	0.9
7	Tooth brushing time	Morning	42	37.2
		Noon	56	49.6
		Night	3	2.7
		Morning and noon	6	5.3
		Morning and night	4	3.5
		Noon and night	2	1.8
8	Movement brushing teeth	Horizontal	64	56.6
		Vertical	28	24.8
		Circular	21	18.6
	Total		113	100.00

**Table 2:** Data on the rate of caries increment in stunted children from the first to the third year.

Increment	Frequency	%
0	4	3.5
1	8	7.1
2	16	14.2
3	23	20.4
4	17	15.0
5	22	19.5
6	11	9.7
7	6	5.3
8	4	3.5
9	2	1.8
Total	113	100

Table 2 shows that the caries increment with a score of three has the highest frequency among all participants, with 23 children (20.4%). Only four children (3.5%) did not show an increase in caries from the first to the third year, while the highest caries increment, totaling nine, is observed in 2 children (1.8%). The distribution of oral hygiene practice and caries increment in stunted children can be seen in Table 3.

**Table 3:** Distribution of oral hygiene practice and caries increment in stunted children.

		Caries Increment				Total	
		Low		High			
		F	%	F	%	F	%
Oral Hygiene Practice	Good	18	15.9	15	13.3	33	29.2
	Poor	50	44.3	30	26.5	80	70.8
Total		68	60.2	45	39.8	113	100

Table 3 shows that most participants (60.2%) experienced a low caries increment from the first to the third year. The table also indicates that most participants (70.8%) had poor oral hygiene practices. Most participants had poor oral hygiene practices with a low caries increment, totaling 50 individuals (44.3%). In comparison, 18 individuals (15.9%) had good oral hygiene practices with a low caries increment.

## Discussion

The chronic malnutrition experienced by stunted children can affect their behaviours in maintaining oral health, including motor skills and the role of parents (6, 7). In this study, most participants (62.8%) reported brushing their teeth daily. This is consistent with the findings of Husna's study, where most respondents (91.4%) reported brushing their teeth daily (21). Brushing teeth is a routine that should be established early to become a habit children practice (22). Furthermore, all participants in this study reported having toothbrushes, which aligns with Mukhbitin's (23) study indicating that all respondents used their toothbrushes. Having a personal toothbrush is essential to prevent the transmission of diseases through toothbrushes used by multiple family members (24).

This study found that most participants (59.3%) reported using fluoride toothpaste when brushing their teeth. This finding is similar to Deinzer's study, where most participants used toothpaste while brushing their teeth (25). Fluoride toothpaste is recommended for effective tooth brushing as it significantly prevents dental caries and plaque (25, 26). Also, decreased saliva flow during sleep can increase the risk of caries, making fluoride toothpaste before bedtime highly recommended (27).

Additionally, most participants (61.9%) showed that they still consumed breast milk or milk with a bottle at night. Therefore, the timing and duration of nutrient intake through breast milk should be considered. Prolonged breastfeeding or formula milk feeding at inappropriate times can contribute to dental caries (28). Similarly, Dianegianty et al. found that most children (86%) still consumed milk before bedtime, which is a risk factor for caries due to the prolonged exposure of teeth to milk (29). During rest or sleep, saliva flow decreases, allowing bacteria to become more active in plaque formation, particularly at night (28).

Regarding the duration of tooth brushing, a significant number of participants (69%) brushed their teeth for less than two minutes. This result aligns with Khan's research, which found that only 4% of participants brushed their teeth for more than two minutes, while the rest brushed for inadequate durations (30). Appropriate tooth brushing habits are defined as brushing twice daily for 120 s, but only a few participants in this study adhered to this practice (25). Short tooth brushing duration may reduce its effectiveness in plaque removal, which can affect the incidence of caries (22). This study also revealed that most participants (88.5%) brushed their teeth only once daily.

In contrast, Ningsih et al. reported different findings regarding tooth brushing frequency, with the majority of participants (79.4%) brushing their teeth twice daily (31). Ideally, tooth brushing should be done at least twice daily, in the morning and before bedtime (22, 27). Nearly half of this study's participants (49.6%) only brushed their teeth in the afternoon. Similar to Diyanata et al.'s study, which showed that nearly half of the participants made mistakes in determining the timing of tooth brushing, such as not brushing after breakfast and before bedtime (32). Brushing teeth at varying times or not at the appropriate times should be avoided from an early age. Although the frequency of tooth brushing is correct, brushing at the wrong time can still pose a high risk of caries in children (29).

Moreover, more than half of the participants (56.6%) brushed their teeth using horizontal movements. Khan's study also found that most samples (55%) used horizontal toothbrushing techniques (30). This situation may be due to the preference for the horizontal technique among individuals who have not received much education on proper tooth brushing techniques, as it is easy to apply (33). In addition, the horizontal technique is considered suitable and easy to learn for children, as their muscle development allows for this technique (22, 33). However, the horizontal technique, which is the most commonly used, is considered less effective in plaque removal and can lead to cervical abrasion, dentin hypersensitivity, or gingival recession when used over a long period (34).

### Oral hygiene practice

One of the goals of oral hygiene practice is to prevent the accumulation of food debris and plaque on the tooth surface, which can lead to dental caries (35). The analysis results indicate that the average score for oral hygiene practice in stunted children in the Sukajadi district is 3.24, which means that, on average, each child only practices correct oral hygiene behaviour in 3 out of 8 aspects. The study findings show that a significant number of participants (70.8%) have poor oral hygiene practices. Similar findings were reported by Diyanata, showing that most respondents had low or poor levels of oral health behaviour (32). This finding can be attributed to parental knowledge. Cognitive knowledge is a crucial domain in shaping behaviour (6).

Children are still under the supervision and dependence of their parents in various aspects, including oral hygiene behaviour. Low parental knowledge can increase the risk of higher caries prevalence in children, as it may not support proper oral hygiene practices. Parents should pay attention to their children's dental hygiene and health from an early age so that children become accustomed to practising good oral hygiene (6). During growth, stunting can harm various aspects, such as fine motor skills, psychomotor skills, neurosensory integration, and disease susceptibility, leading to reduced productivity (7). Providing training to children can positively contribute to the development of motor skills and excellent motor skills (22). The role of parents and their level of knowledge significantly influence efforts to prevent plaque accumulation and dental caries in children, reducing the risk of caries occurrence. Therefore, Indonesia faces a significant demand to provide dental care programs to improve the oral health of the entire population without exception (6).

### Caries increment

The following study regarding the increase in dental caries reveals that the average caries increment in stunted children in the Sukajadi district from the first to the third year is 4.94, indicating that each child experienced an increase in carious teeth by four to five. The analysis by Deinzer indicates that many respondents still have improper tooth brushing behaviour, resulting in a high prevalence of caries (25). Based on the data on the distribution of oral hygiene practice and caries increment in stunted children, a significant portion of the participants (44.3%) had low caries increment but poor oral hygiene practices. The data also indicate that there is still an increase in caries prevalence among the group with good oral hygiene practices, although compared to those with poor oral hygiene practices, the number of caries increments is higher. There is a tendency for caries in children with improper tooth brushing behaviour, although other underlying factors contribute to dental caries. Stunted children may understand the knowledge and behaviours related to oral health but struggle to implement these actions daily. Husna's study in 2019 shows that oral health behaviour in stunted children is still low (21). One factor affecting the behaviour of stunted children is their tendency to have suboptimal intelligence due to chronic malnutrition. Chronic malnutrition can lead to metabolic changes in the brain, resulting in lower cognitive function (36).

Various factors may influence participants with good oral hygiene but high caries increment. Physiological factors in stunted children, such as the vulnerability of teeth to caries due to long-term nutritional deficiencies, can play a role. In this study, in addition to poor oral hygiene practices, the increased caries prevalence among children may also be due to abnormal saliva acidity levels and dietary habits, such as the consumption of cariogenic foods that children, such as chocolate, biscuits, ice cream, candies, and others commonly like (33). Each risk factor strengthens the occurrence of dental caries. The high number of participants with significant caries increment can be attributed to various factors, including the improper oral hygiene practices observed in this study. Many practices, such as consuming breast milk or milk with a bottle at night, brushing teeth for less than 2 min, brushing teeth only once a day instead of in the morning and at night, or using improper tooth brushing techniques, are still prevalent. Biological factors in stunted children resulting from insufficient nutrient intake can also contribute to the increased caries prevalence. Other factors may include socioeconomic factors and low awareness of daily oral hygiene practices (28, 29).

Most participants in this study had poor oral hygiene practices, but their caries increment was relatively low. These findings indicate that there is still an increase in caries prevalence, although the numbers are considered low. Oral hygiene practices are just one of many factors that can influence the incidence of dental caries. The researchers assume that many other factors affect the population in that area, although they were not studied in this research. Some of these factors include dietary patterns and dental visit routines. Access to information and facilities regarding these factors is relatively easy for the population in Bandung, which can contribute to preventing the increase in dental caries through monitoring healthy eating habits and regular dental visits for children accompanied by their parents.

Dental health promotion programs have been developed using various communication tools and media (27). Poor oral hygiene behaviour can lead to oral health problems. If dental and oral health issues in stunted children are not addressed, it can exacerbate malnutrition and reduce the quality of life for these children. Parents need to pay extra attention to maintaining and caring for stunted children's dental and oral health compared to well-nourished children. The importance of parental involvement can be seen in how they guide, direct, teach, and motivate children to maintain their dental and oral health (37). Therefore, all dental healthcare professionals, public health practitioners, and related industries must participate in reaching out to the community in evaluating and developing health programs. These programs could include educating adolescents about the importance of selecting nutritious foods to optimize their overall health and providing information on fluoride as a preventive measure against high caries prevalence.

The limitations of this study are that it relied on existing data, so adjustments such as the length of the caries examination period or the caries index used could not be made. This study only provided a descriptive overview and did not analyze the relationships between variables. Recommendations for further research include investigating the relationships between different aspects of oral hygiene practices and caries increment in stunted children. It is also suggested that parents monitor essential aspects of their children's oral health behaviour regularly and modify toothbrushing activities to make them enjoyable and tailored to each child's character.

## Conclusion

The study concludes a high prevalence of poor oral hygiene practices among stunted children in Sukajadi Bandung. Despite most participants having regular toothbrushing habits, the duration, frequency, and technique of toothbrushing are often inadequate. Additionally, many children still consume breast milk or milk with a bottle at night, which increases the risk of dental caries. The study also found a significant increase in caries increment among stunted children, indicating a need for improved oral hygiene practices and preventive measures.

The findings highlight the importance of parental involvement and education in promoting good oral hygiene practices among stunted children. Parents are crucial in guiding and motivating their children to maintain proper dental and oral health habits. Therefore, it is recommended to implement comprehensive dental health promotion programs that educate parents and raise awareness about the importance of appropriate toothbrushing techniques, regular dental visits, and a healthy diet.

The study emphasizes the need for further research to explore the relationships between different aspects of oral hygiene practices and caries increment in stunted children. It is also suggested that future studies investigate the impact of various factors, such as socioeconomic status and nutritional intake, on oral health outcomes in this population.

Overall, the study underscores the importance of addressing oral health issues among stunted children to prevent further deterioration of their overall health and improve their quality of life. Effective oral health interventions, parental involvement, and community-based programs are essential in reducing the prevalence of dental caries and promoting better oral health outcomes in stunted children.

## Data Availability

The original contributions presented in the study are included in the article/Supplementary Material, further inquiries can be directed to the corresponding author.
